# Brain pericytes serve as microglia-generating multipotent vascular stem cells following ischemic stroke

**DOI:** 10.1186/s12974-016-0523-9

**Published:** 2016-03-07

**Authors:** Rika Sakuma, Maiko Kawahara, Akiko Nakano-Doi, Ai Takahashi, Yasue Tanaka, Aya Narita, Sachi Kuwahara-Otani, Tetsu Hayakawa, Hideshi Yagi, Tomohiro Matsuyama, Takayuki Nakagomi

**Affiliations:** Institute for Advanced Medical Sciences, Hyogo College of Medicine, 1-1 Mukogawacho, Nishinomiya, Hyogo 663-8501 Japan; Graduate School of Science and Technology, Kwansei Gakuin University, 2-1 Gakuen, Sanda, Hyogo 669-1337 Japan; Department of Neurosurgery, Hyogo College of Medicine, 1-1 Mukogawacho, Nishinomiya, Hyogo 663-8501 Japan; Laboratory of Tumor Immunology and Cell Therapy, Hyogo College of Medicine, 1-1 Mukogawacho, Nishinomiya, Hyogo 663-8501 Japan; Department of Anatomy and Neuroscience, Hyogo College of Medicine, 1-1 Mukogawacho, Nishinomiya, Hyogo 663-8501 Japan

**Keywords:** Ischemia, Stroke, Vascular stem cells, Microglia, Pericytes

## Abstract

**Background:**

Microglia are the resident macrophage population of the central nervous system (CNS) and play essential roles, particularly in inflammation-mediated pathological conditions such as ischemic stroke. Increasing evidence shows that the population of vascular cells located around the blood vessels, rather than circulating cells, harbor stem cells and that these resident vascular stem cells (VSCs) are the likely source of some microglia. However, the precise traits and origins of these cells under pathological CNS conditions remain unclear.

**Methods:**

In this study, we used a mouse model of cerebral infarction to investigate whether reactive pericytes (PCs) acquire microglia-producing VSC activity following ischemia.

**Results:**

We demonstrated the localization of ionized calcium-binding adaptor molecule 1 (Iba1)-expressing microglia to perivascular regions within ischemic areas. These cells expressed platelet-derived growth factor receptor-β (PDGFRβ), a hallmark of vascular PCs. PDGFRβ^+^ PCs isolated from ischemic, but not non-ischemic, areas expressed stem/undifferentiated cell markers and subsequently differentiated into various cell types, including microglia-like cells with phagocytic capacity.

**Conclusions:**

The study results suggest that vascular PCs acquire multipotent VSC activity under pathological conditions and may thus be a novel source of microglia.

**Electronic supplementary material:**

The online version of this article (doi:10.1186/s12974-016-0523-9) contains supplementary material, which is available to authorized users.

## Background

Microglia, a glial cell subtype, comprise the resident macrophage population located within the central nervous system (CNS). The precise origin of microglia has long remained the subject of debate [1, 2]. Previous studies have demonstrated that microglia originate from progenitor cells in the embryonic yolk sac during early development and that embryonically derived microglia self-maintain until adulthood under normal conditions [3, 4]. Nevertheless, it remains unclear whether these cells can continuously produce microglia in the adult CNS, even under pathological conditions. It has been proposed that some microglia originate from bone-marrow-derived hematopoietic cells or circulating monocytes [5–7]. However, it remains controversial whether new microglia do indeed originate from bone marrow cells [8], suggesting the potential for other sources of microglia within the adult CNS.

Mounting evidence suggests that progenitor cells localized to the adventitia (adventitial progenitor cells (APCs)) around the blood vessels may serve as multipotent resident vascular stem cells (VSCs) [9] that contribute to vasculogenesis [10–12]. A recent study by Psaltis and colleagues demonstrated that macrophage progenitors can derive from APCs located in the adult murine aorta [13]. In addition to the APCs near larger vessels, vascular pericytes (PCs) located around capillaries are also strong candidate sources for the VSC population [9, 14]. PCs exhibit the potential for differentiation into multiple different cell populations, including neural cells, adipocytes, chondroblasts, and osteoblasts [15, 16]. Although it remains controversial whether PCs can produce microglia [17–19], we recently demonstrated that PCs acquire multipotent stem cell activity in response to brain injuries such as ischemia/hypoxia and that these reactive PCs can differentiate into various lineages, including the neural and vasculogenic lineages [20]. In addition, brain multipotent stem cells exhibit microglia-like cell phenotypes [21, 22] and microglia have been described as arising from meningeal cells [2, 23]. We demonstrated that substantial quantities of multipotent PCs were derived from the latter cells following ischemic stroke [20, 24–27]. These findings led us to hypothesize that resident microglia might originate from ischemia-induced multipotent PCs following CNS injury.

In this study, we used a mouse model of cerebral infarction to investigate whether reactive PCs develop the traits of microglia-producing VSCs following ischemia.

## Methods

### Induction of focal cerebral ischemia

The Animal Care Committee of the Hyogo College of Medicine approved all experimental procedures (license number: 12-064). Six-week-old male CB-17/Icr-+/+Jcl mice (CB-17 mice; Clea Japan Inc., Tokyo, Japan) were subjected to cerebral ischemia as described previously [20, 25, 28–30]. Permanent focal cerebral ischemia was produced by ligation and interruption of the distal portion of the left middle cerebral artery (MCA) [20, 25, 28–30]. Under halothane inhalation, the left MCA was isolated, electrocauterized, and disconnected just distal to the point where it crosses the olfactory tract (the distal M1 portion).

### Isolation of PDGFRβ^+^ pericytes following ischemia

Post-ischemia PCs (iPCs) were extracted from post-stroke CB-17 mice as described previously [20, 25, 28–30]. Briefly, mice were deeply anesthetized with sodium pentobarbital (50 mg/kg) on post-stroke day 3. The post-ischemic areas, which contained leptomeninges harboring abundant iPCs [25, 26], were carefully removed under a microscope (Carton, Pathum Thani, Thailand). The removed tissues were mechanically dissociated by passage through 18-, 23-, and 27-gauge needles to create a single-cell suspension. The resulting cell suspensions were incubated with adherent cultures in DMEM/F12 medium (Invitrogen, Carlsbad, CA, USA) containing fibroblast growth factor-basic (bFGF 20 ng/mL; Peprotech, Rocky Hill, NJ, USA), epidermal growth factor (EGF 20 ng/mL; Peprotech), 1 % N2 supplement (Invitrogen), and 2 % fetal bovine serum (FBS). On incubation day 7, the expanded PCs were subjected to magnetic cell sorting (MACS) as described previously [20, 25]. The MACS-sorted platelet-derived growth factor receptor-β (PDGFRβ^+^) iPCs were reincubated with floating cultures in neural-conditioned medium (NCM; DMEM/F12, EGF, FGF-2, and N2) [25, 28–30]. After incubation, PDGFRβ^+^ iPCs that formed clusters were subjected to immunohistochemistry, cell differentiation, electron microscopy, reverse transcriptase-polymerase chain reaction (RT-PCR), and phagocytosis analyses.

### Immunohistochemistry

Coronal brain sections were prepared and subjected to immunohistochemistry as described previously [25, 28–30]. Briefly, mice were anesthetized with sodium pentobarbital and perfused transcardially with 4 % paraformaldehyde on days 3, 5, and 7 after stroke. The perfused brains were removed, cryoprotected in 30 % sucrose, and sectioned on a cryostat. Samples were labeled with antibodies against ionized calcium binding adaptor molecule 1 (Iba1; Abcam, Cambridge, UK), PDGFRβ (Santa Cruz Biotechnology, Santa Cruz, CA, USA), CD31 (BD Pharmingen, San Diego, CA, USA), α-smooth muscle actin (αSMA; Millipore, Temecula, CA, USA), nestin (Millipore), and Sox2 (Millipore). PDGFRβ^+^ iPC-derived clusters were fixed in paraformaldehyde (4 %) and cut on a cryostat, and the resulting tissue sections were subjected to immunohistochemistry. Samples were labeled with antibodies against nestin (Millipore), PDGFRβ (Santa Cruz Biotechnology), neural/glial antigen 2 (NG2; EMD/Millipore, Billerica, MA, USA), αSMA (LifeSpan Biosciences, Seattle, WA, USA), Iba1 (Abcam), and CD68 (Abcam). Bound primary antibodies were visualized using Alexa Fluor 488- or 555-conjugated secondary antibodies (Molecular Probes, Eugene, OR, USA). Nuclei were stained with 4′,6-diamidino-2-phenylindole (DAPI; Kirkegaard & Perry Laboratories, Inc., Gaithersburg, MD, USA). Brain sections were imaged using a confocal laser microscope (LSM780; Carl Zeiss, Jena, Germany). For negative control immunohistochemistry, the primary antibodies were omitted and no staining was confirmed. In this study, we defined the “ischemic core” as the internal area within the “border of the post-stroke area” and the “peri-ischemic area” as the external (non-ischemic) regions within 200 μm of the “border of the post-stroke area.” The numbers of positive cells were analyzed using Image J software (a total of 15 data points, 5 points/section (*n* = 3)) and subjected to a semi-quantitative analysis as described [30, 31]. Other methods for immunohistochemistry are available in Additional file 1.

### Cell differentiation

To induce neural differentiation, PDGFRβ^+^ iPC clusters were incubated on poly-l-lysine-coated glass coverslips for 7 days in neurobasal medium (Invitrogen, Carlsbad, CA, USA) supplemented with B-27 (Invitrogen) and all-trans retinoic acid (0.2 μM; Sigma, St. Louis, MO, USA) [20]. Differentiated cells were labeled with antibodies against Iba1 (Abcam), CD11b (BD Pharmingen), major histocompatibility complex (MHC) class 1 (Abcam), and Tuj1 (Stem Cell Technologies, Vancouver, BC, Canada). To induce osteoblastic or adipogenic differentiation, PDGFRβ^+^ iPCs were cultured in osteogenic or adipogenic differentiation medium, respectively, according to the manufacturer’s protocol (SC 010; R&D systems, Minneapolis, MN, USA). The resulting differentiated cells were labeled with antibodies against osteopontin (Santa Cruz Biotechnology) or fatty acid binding protein 4 (FABP4) (R&D Systems), respectively. Alternatively, PDGFRβ^+^ iPCs incubated in adipogenic differentiation medium were stained with the lipid-specific dye Oil Red O as described previously [32].

### Electron microscopy

Differentiated cell clusters were sequentially fixed in a 1 % glutaraldehyde and 1 % paraformaldehyde solution of 0.1-M phosphate buffer, pH 7.4, for 1 h. After a brief rinse in phosphate buffer, the cell clusters were post-fixed with 2 % OsO_4_ in phosphate buffer for 2 h, followed by methanol dehydration and embedding between Aclar films (Nisshin EM, Tokyo, Japan) with Epon812. Ultrathin sections were subsequently cut and collected on Formvar-coated single-slot grids. The sections were stained with uranyl acetate and Reynolds’ solution and examined with a JEOL 1220EX transmission electron microscope (JEOL GmbH, Freising, Germany).

### OGD treatment

Commercially available adult normal mouse brain PCs (PC-N; #M1200, Scien Cell Research Laboratories, Carlsbad, CA, USA) were incubated under oxygen/glucose deprivation (OGD) (PC-OGD). In brief, the cells (5.0 × 10^4^ cells/well) were placed on 12-well culture plates (Iwaki, Tokyo, Japan) in pericyte growth medium (Scien Cell Research Laboratories). One day later, the medium was removed and replaced with glucose-free DMEM and FBS (2 %). PC-OGD were then incubated under hypoxia (1 % O_2_) for 7 days using a hypoxia-inducing system (Bionix, SUGIYAMA-GEN, Tokyo, Japan) as described previously [20].

### RT-PCR

Total RNA was extracted from PDGFRβ^+^ iPCs, PC-N, and PC-OGD using an RNeasy Micro Kit (Qiagen, Hilden, Germany). Complementary DNAs (cDNAs) were amplified according to the manufacturer’s protocols as described previously [20, 25, 28]. The following primer sequences were used in these reactions: PDGFRβ forward, 5**′**-ACAATTCCGTGCCGAGTGACAG-3**′** and PDGFRβ reverse, 5**′**-AAAAGTACCAGTGAAACCTCGCTG-3**′** (amplicon size, 114 bp); NG2 forward, 5**′**-ATGCTTCTCAGCCCGGGACA-3**′** and NG2 reverse, 5**′**-GGTTGCGGCCATTGAGAATG-3**′** (amplicon size, 541 bp); αSMA forward, 5**′**-GGACGTACAACTGGTATTGTGC-3**′** and αSMA reverse, 5**′**-TCGGCAGTAGTCACGAAGGA-3**′** (amplicon size, 179 bp); nestin forward, 5**′**-CACTAGAAAGCAGGAACCAG-3**′** and nestin reverse, 5**′**-AGATGGTTCACAATCCTCTG-3**′** (amplicon size, 307 bp); c-myc forward, 5**′**-ATGCCCCTCAACGTGAACTTC-3**′** and c-myc reverse, 5**′**-CGCAACATAGGATGGAGAGCA-3**′** (amplicon size, 228 bp); Klf4 forward, 5**′**-GTGCCCCGACTAACCGTTG-3**′** and Klf4 reverse, 5**′**-GTCGTTGAACTCCTCGGTCT-3**′** (amplicon size, 185 bp); Sox2 forward, 5**′**-TTGGGAGGGGTGCAAAAAGA-3**′** and Sox2 reverse, 5**′**-CCTGCGAAGCGCCTAACGTA-3**′** (amplicon size, 312 bp); Iba1 forward, 5**′**-GGATTTGCAGGGAGGAAAAG-3**′** and Iba1 reverse, 5**′**-TGGGATCATCGAGGAATTG-3**′** (amplicon size, 92 bp); CD11b forward, 5**′**-GGGAGGACAAAAACTGCCTCA-3**′** and CD11b reverse, 5**′**-ACAACTAGGATCTTCGCAGCAT-3**′** (amplicon size, 98 bp); and β-actin forward, 5**′**-GCTCGTCGTCGACAAGGGCTC-3**′** and β-actin reverse, 5**′**-CAAACATGATCTGGGTCATCTTCTC-3**′** (amplicon size, 353 bp).

### Phagocytosis assay

To determine whether post-ischemia reactive brain PCs could potentially give rise to functional microglia capable of phagocytosis, PDGFRβ^+^ iPCs were induced to differentiate and then subjected to treatment with a Latex Beads-Rabbit IgG-FITC solution, a component of a phagocytosis assay kit, according to the manufacturer’s protocols (Cayman Chemical, Ann Arbor, MI, USA).

## Results

### Localization and characterization of microglia following ischemic stroke

We first examined the localization of Iba1-expressing microglia following ischemia. In sham-operated mice, Iba1^+^ microglia were observed in the MCA areas of the CNS. These resting microglia displayed a ramified morphology (Additional file 2: Figure S1A–C). On post-stroke day 3, Iba1^+^ microglia accumulated predominantly in the peri-ischemic areas (Additional file 2: Figure S1D–F). Although some cells still showed a ramified morphology, this population had decreased by post-ischemic days 5 (Additional file 2: Figure S1G–I) and 7 (Additional file 2: Figure S1J–L). In contrast, ameboid-like activated microglia frequently appeared in the peri-ischemic areas by post-stroke day 5 (Additional file 2: Figure S1G–I) and further increased at post-ischemic day 7 (Additional file 2: Figure S1J–L). Iba1^+^ cell numbers within the ischemic core and peri-ischemic areas were determined following ischemia (Additional file 2: Figure S1M), with the populations of ramified- and ameboid-like Iba1^+^ microglia assessed in both areas (Additional file 2: Figure S1N).

### Iba1 expression by PDGFRβ^+^-reactive brain PCs

**Fig. 1:**
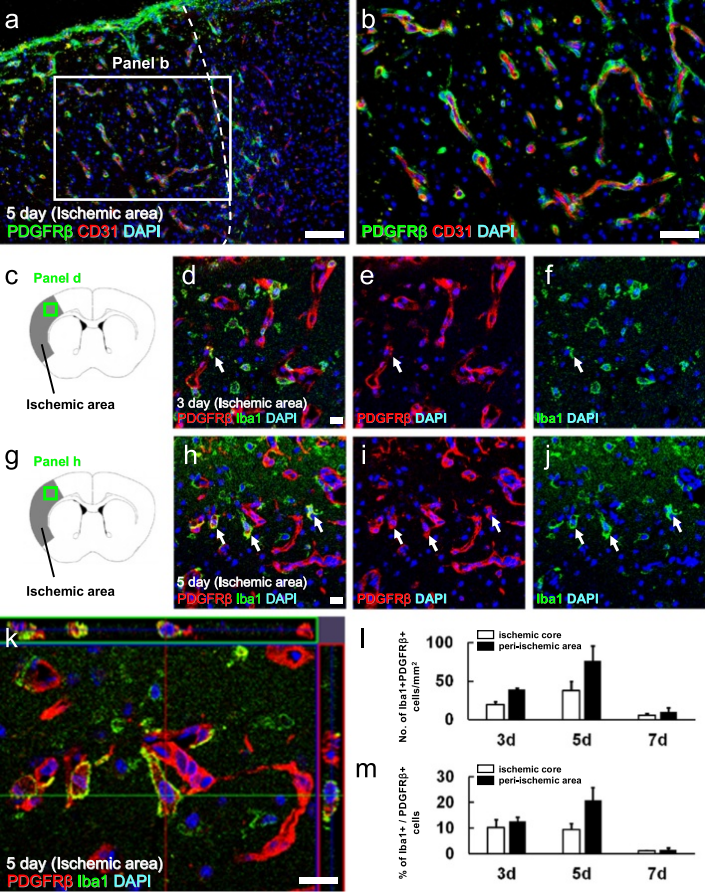
Brain PDGFRβ^+^ PCs express Iba1 following ischemia. At the ischemic site, PDGFRβ^+^ expression was specifically observed in PCs located near CD31^+^ endothelial cells (PDGFRβ (**a**, **b** *green*), CD31 (**a**, **b** *red*), DAPI (**a**, **b** *blue*)). On day 3 post-stroke, only a few PDGFRβ^+^ iPCs expressed Iba1 (**c**–**f**) (PDGFRβ (**d**, **e** *red*), Iba1 (**d**, **f** *green*), DAPI (**d**–**f** *blue*)) (*arrow*). However, some Iba1^+^PDGFRβ^+^ iPCs were observed in the ischemic areas on post-stroke day 5 (**g**–**k**) (PDGFRβ (**h**, **i**, **k**: *red*), Iba1 (**h**, **j**, **k** *green*), DAPI (**h**–**k** *blue*)) (*arrows*). The numbers of Iba1^+^ and PDGFRβ^+^ cells (**l**) and populations of Iba1^+^ cells relative to those of the PDGFRβ^+^ cells (**m**) localized to the ischemic core and peri-ischemic areas are shown. *Scale bars* = 100 μm (**a**), 50 μm (**b**), and 20 μm (**d**, **h**, **k**). *Iba1* ionized calcium binding adaptor molecule 1, *PDGFRβ* platelet-derived growth factor receptor-β

To determine whether microglia originate, at least in part, from reactive PCs, we first investigated the localization of PDGFRβ^+^ cells following ischemic stroke. Immunohistochemistry showed that PDGFRβ^+^ cells localized near CD31^+^ endothelial cells in and around the ischemic areas (Fig. 1a, b). Morphologically, PDGFRβ^+^ cells extend long cell processes across the surface of endothelial cells, a characteristic feature also exhibited by PCs [33]. This finding confirmed the expression of PDGFRβ in reactive PCs following ischemia. We then investigated whether PDGFRβ^+^ PCs express Iba1 following ischemic stroke. On post-stroke day 3, only a small portion of PDGFRβ^+^ cells expressed Iba1 (Fig. 1c–f). However, this number began to increase on post-stroke day 5 (Fig. 1g–k), suggesting that PDGFRβ^+^ iPCs could generate microglia. The number of Iba1 and PDGFRβ double-positive cells (Fig. 1l) and the populations of Iba1^+^ cells relative to PDGFRβ^+^ cells (Fig. 1m) were analyzed at post-stroke days 3, 5, and 7 within the ischemic core and peri-ischemic areas.

**Fig. 2:**
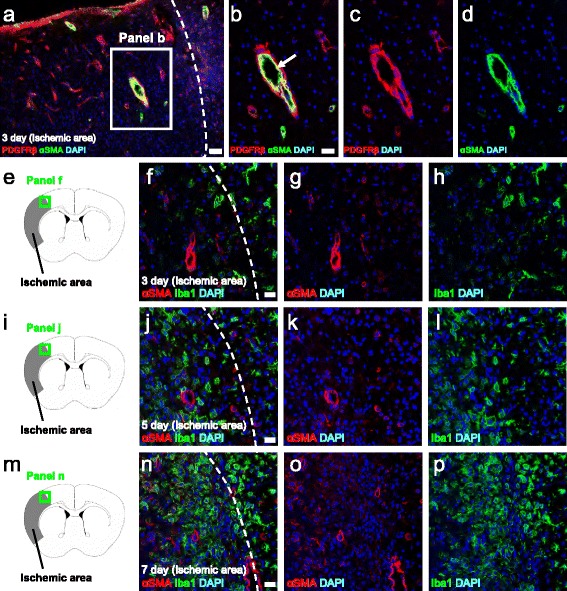
Brain αSMA^+^ cells rarely express Iba1 following ischemia. Immunohistochemistry showed that αSMA expression was predominantly observed in the smooth muscle cells located around large vessels that also expressed PDGFRβ (PDGFRβ (**a**–**c** *red*), αSMA (**a**, **b**, **d** *green*), DAPI (**a**–**d** *blue*)). However, following ischemia, Ibal^+^ cells rarely expressed αSMA at post-stroke day 3 (**e**–**h**), 5 (**i**–**l**), and 7 (**m**–**p**) (αSMA (**f**, **g**, **j**, **k**, **n**, **o** *red*), Iba1 (**f**, **h**, **j**, **l**, **n**, **p** *green*), and DAPI (**f**–**h**, **j**–**l**, **n**–**p** *blue*)). This pattern suggested that microglia originate in part from perivascular cells and in particular from PCs rather than smooth muscle cells. *Scale bars* = 50 μm (**a**) and 20 μm (**b**, **f**, **j**, **n**) *Iba1* ionized calcium binding adaptor molecule 1, *PDGFRβ* platelet-derived growth factor receptor-β

We further investigated whether PDGFRβ^+^ cells within the ischemic areas expressed αSMA. The latter protein is known to be expressed in perivascular cells and, in particular, in smooth muscle cells located around large vessels, rather than in the PCs that surround capillaries [34]. Consistent with the previous report, αSMA cells were predominantly observed in smooth muscle cells and they also expressed PDGFRβ (Fig. 2a–d). This finding suggests that PDGFRβ was expressed not only in PCs but also in smooth muscle cells. Thus, we examined whether Ibal^+^ cells express αSMA following ischemia. The results indicated that Ibal^+^ cells rarely expressed αSMA on post-stroke days 3 (Fig. 2e–h), 5 (Fig. 2i–l), and 7 (Fig. 2m–p), indicating that microglia originate in part from perivascular cells and in particular from PCs rather than smooth muscle cells.

We next investigated whether PDGFRβ^+^ iPCs express the microglial markers other than Iba1. Immunohistochemistry at post-stroke day 3 showed that some PDGFRβ^+^ cells co-express the microglial marker CD206 [35] (Additional file 3: Figure S2A–D). We then investigated whether PDGFRβ^+^ cells express CD68, which is generally known to be expressed by perivascular macrophages rather than by microglia [35, 36]. On post-stroke day 3, CD68^+^ cells were only rarely observed within the ischemic core and the peri-ischemic areas (Additional file 4: Figure S3A–D). CD68^+^ cells were observed at these areas on post-stroke day 5 (Additional file 4: Figure S3E–H) and 7 (Additional file 4: Figure S3I–L) and some of them expressed Iba1 (Additional file 4: Figure S3E–L). However, there were fewer CD68^+^ cells than Iba1^+^ cells within these regions. In addition, PDGFRβ^+^ cells at the ischemic core and peri-ischemic areas rarely express CD68 on post-stroke days 5 (Additional file 4: Figure S3M–P) and 7 (Additional file 4: Figure S3Q–T). These findings were consistent with a previous report showing that regulator of G-protein signaling 5 (RGS5)^+^ PCs following ischemic stroke express Iba1 but not CD68 [36]. Together, these results suggest that Iba1^+^ microglia that appear following ischemia are most likely not derived from perivascular macrophages.

### Post-ischemic brain PCs express the stem cell markers

**Fig. 3:**
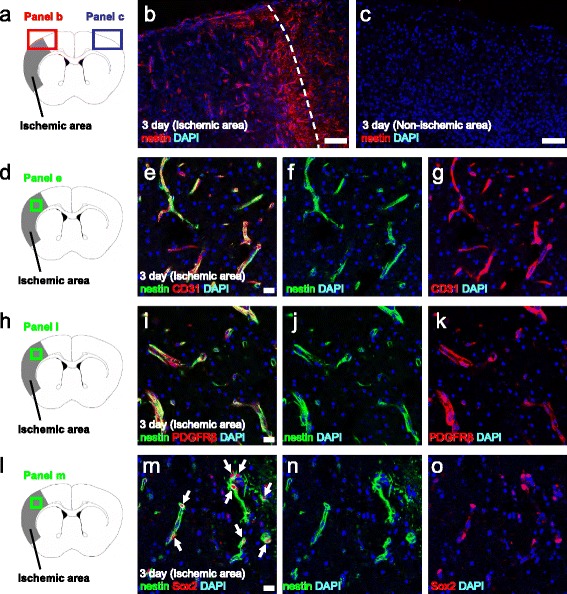
Brain PCs express the stem cell markers following ischemia. Brain PCs expressed the neural stem cell marker nestin on post-stroke day 3 (**a**–**c**). Although nestin was highly expressed in ischemic areas (**a**, **b**), it was rarely observed in non-ischemic areas (**a**, **c**) (nestin (**b**, **c** *red*), DAPI (**b**, **c** *blue*)). Nestin^+^ cells within the ischemic core were localized near CD31^+^ endothelial cells (**d**-**g**) (nestin (**e**, **f** *green*), CD31 (**e**, **g** *red*), DAPI (**e**–**g** *blue*)) and the majority also expressed PDGFRβ (**h**-**k**) (nestin (**i**, **j** *green*), PDGFRβ (**i**, **k** *red*), DAPI (**i**–**k** *blue*)). In addition, nestin^+^ cells located within ischemic areas also expressed the stem cell marker Sox2 (**l**-**o**) (nestin (**m**, **n** *green*), Sox2 (**m**, **o** *red*), DAPI (**m**–**o** *blue*)). *Scale bars* = 100 μm (**b**, **c**) and 20 μm (**e**, **i**, **m**) *PDGFRβ* platelet-derived growth factor receptor-β

We recently demonstrated that reactive PCs acquire multipotent stem cell potential following ischemia [20]. Thus, we next examined whether PDGFRβ^+^ PCs express the stem cell marker nestin following ischemic stroke. Although nestin was observed within ischemic areas (Fig. 3a, b), it was not expressed in non-ischemic areas on post-stroke day 3 (Fig. 3a, c). In addition, nestin^+^ cells within ischemic areas largely (92.5 %) localized near CD31^+^ endothelial cells (Fig. 3d–g) and they frequently (61.1 %) expressed PDGFRβ (Fig. 3h–k). Furthermore, nestin^+^ cells within ischemic areas expressed the stem cell marker Sox2 in the nucleus (Fig. 3l–o), confirming that nestin^+^ cells have the traits of stem cells. These results suggest that PDGFRβ^+^ PCs within ischemic areas develop stemness following ischemia.

### Brain PCs acquire multipotent VSC activity following ischemia

**Fig. 4:**
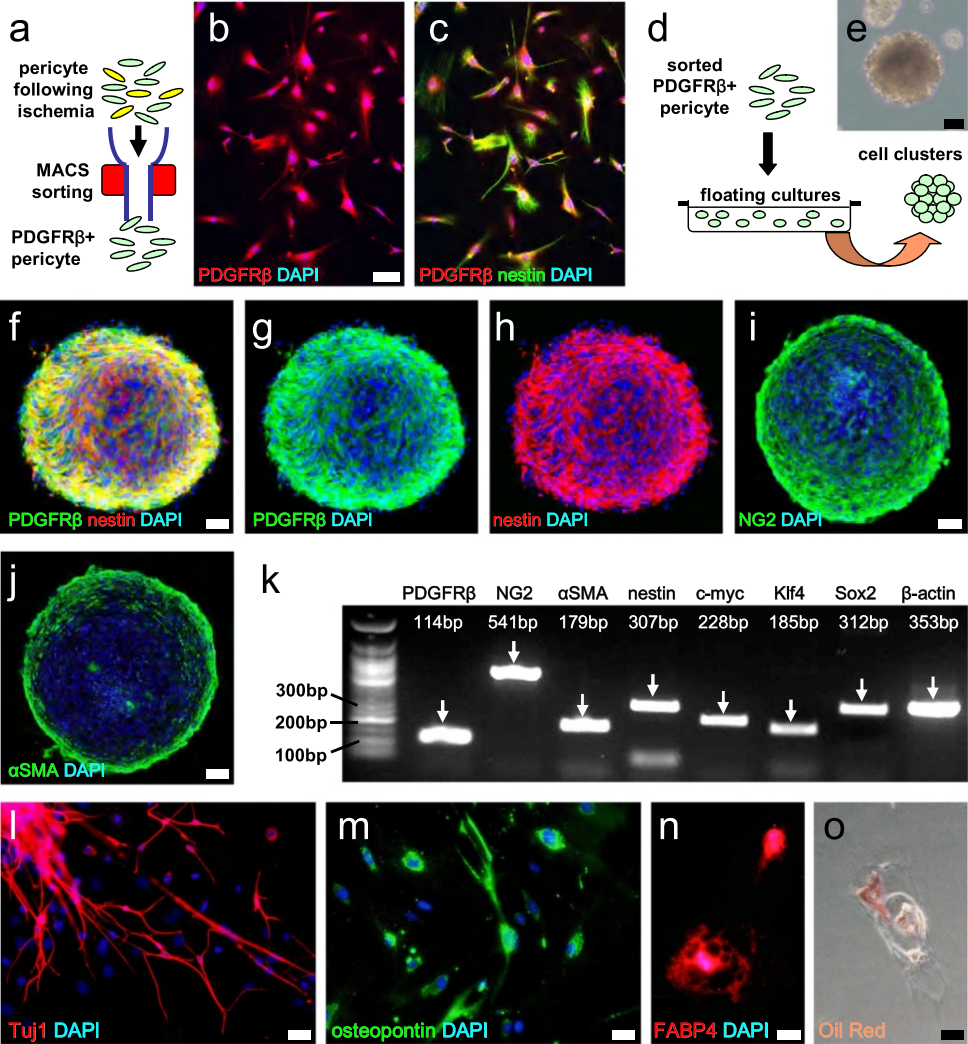
Brain PCs serve as multipotent VSCs following ischemia. Cells were isolated from ischemic areas, and PDGFRβ^+^ iPCs were selectively collected using MACS (**a**). By immunohistochemistry, MACS-sorted cells expressed both PDGFRβ and nestin (PDGFRβ (**b**, **c** *red*), nestin (**c** *green*), DAPI (**b**, **c** *blue*)). After incubation in a floating culture (**d**), PDGFRβ^+^ iPCs formed clusters (**e**). The cells in these clusters expressed nestin (PDGFRβ (**f**, **g** *green*), nestin (**f**, **h** *red*), DAPI (**f**–**h** *blue*)) and PC cell marker proteins that included NG2 (NG2 (**i** *green*), DAPI (**i**: *blue*)), and αSMA (αSMA (**j** *green*), DAPI (**j** *blue*)). PCR analysis confirmed that the clusters expressed PC markers (PDGFRβ, NG2, and αSMA). In addition, these cells expressed various stem and undifferentiated cell markers, including nestin, c-myc, Klf4, and Sox2 (**k**). The PDGFRβ^+^ iPCs subsequently differentiated into various cell types, including Tuj1^+^ neuronal cells (**l**), osteopontin^+^ osteoblasts (**m**), FABP4^+^ (**n**), and Oil red^+^ adipocytes (**o**). *Scale bars* = 50 μm (**b**, **e**, **f**, **i**, **j**), and 20 μm (**l**, **m**, **n**, **o**) *FABP4* fatty acid binding protein 4, *MACS*, magnetic cell sorting, *PDGFRβ* platelet-derived growth factor receptor-β, *VSCs* vascular stem cells

To confirm that PDGFRβ^+^ PCs acquire multipotent VSC activity in response to ischemia, PDGFRβ^+^ cells displaying PC characteristics were selectively collected from ischemic areas using a MACS-based procedure (Fig. 4a) as described previously [20]. Immunohistochemistry revealed that all sorted cells expressed PDGFRβ (Fig. 4b), confirming the successful collection of PDGFRβ^+^ cells. In addition, some PDGFRβ^+^ cells co-expressed nestin (Fig. 4c), indicating that PDGFRβ^+^ iPCs have stem cell characteristics. To confirm this observation, MACS-sorted PDGFRβ^+^ iPCs were incubated in medium under floating culture conditions (Fig. 4d). Following incubation, these cells formed clusters (Fig. 4e) that were positive for nestin (Fig. 4f–h), as well as various PC markers such as NG2 (Fig. 4i) and αSMA (Fig. 4j). PCR analysis also revealed that in addition to PC marker (e.g., PDGFRβ, NG2, and αSMA) expression, the clustered cells also expressed various stem and undifferentiated cell markers, including nestin, c-myc, Klf4, and Sox2 (Fig. 4k). After differentiation, PDGFRβ^+^ iPCs revealed their potential for differentiation into various cell populations, including Tuj1^+^ neuronal cells (Fig. 4l), osteopontin^+^ osteoblasts (Fig. 4m), and FABP4^+^ (Fig. 4n) and Oil Red^+^ adipocytes (Fig. 4o). However, we could not isolate these cells from non-ischemic areas as previously described [20, 25, 28]. These results indicate that reactive PDGFRβ^+^ iPCs harbor multi-differentiation potential and can function as VSCs.

### Brain PCs have the potential to generate microglia following ischemia

**Fig. 5:**
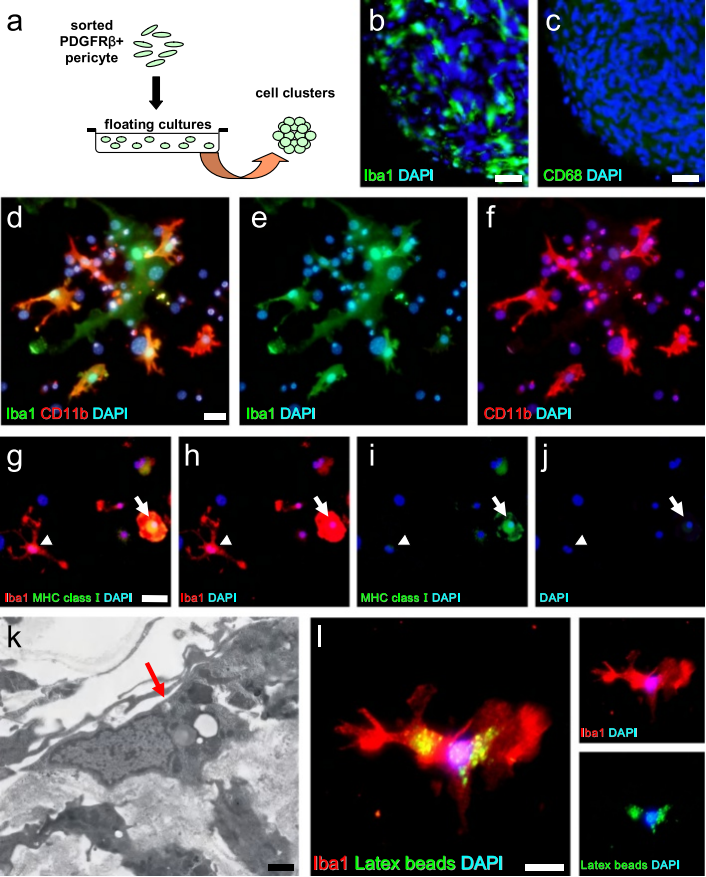
Brain PCs can generate microglia following ischemia. Some of the clustered MACS-sorted PDGFRβ^+^ iPCs (**a**) expressed Iba1 (**b**) (Iba1 (**b** *green*), DAPI (**b** *blue*)), but not CD68 (**c**) (CD68 (**c** *green*), DAPI (**c** blue)). The cell clusters differentiated into Iba1^+^ and CD11b^+^ microglia (Iba1 (**d**, **e** *green*), CD11b (**d**, **f** *red*), DAPI (**d**–**f** blue)). The differentiated Iba1^+^ ameboid-like microglia, but not the ramified-like microglia, expressed the MHC class 1 protein (*arrow* and *arrowhead*, respectively) (Iba1 (**g**, **h** red), MHC class I (**g**, **i** *green*), DAPI (**g**–**j** *blue*)). Electron microscopy revealed that differentiated cells have microglial traits and show increased electron density at the cytoplasmic periphery (**k**, *arrow*). Iba1^+^ ameboid-like microglia were able to phagocytose latex beads (Iba1 (**l** *red*), latex beads (**l** *green*), DAPI (**l** *blue*)). *Scale bars* = 50 μm (**b**, **c**), 20 μm (**d**, **g**), 1 μm (**k**), and 10 μm (**l**) *Iba1* ionized calcium binding adaptor molecule 1, *MACS* magnetic cell sorting, *MHC* major histocompatibility complex, *PDGFRβ* platelet-derived growth factor receptor-β

To confirm that reactive brain PCs can develop into microglia following ischemia, MACS-sorted, clustered PDGFRβ^+^ iPCs (Fig. 5a) were induced to differentiate. To exclude potential contamination with perivascular macrophages, the spheres were immunostained with CD68. The cell clusters expressed Iba1 (Fig. 5b) but not CD68 (Fig. 5c), indicating that they did not contain perivascular macrophages. In addition, the differentiated cells expressed Iba1 and some also expressed CD11b, an integrin receptor that serves as a microglial marker (Fig. 5d–f). Morphologically, the differentiated Iba1^+^ cells displayed the traits of both ramified and ameboid microglia (Fig. 5g, h, j). Iba1^+^ ameboid-like microglia, but not ramified-like microglia, expressed MHC class 1 (Fig. 5g, i, j). Electron microscopy revealed that these cells also showed other microglial traits, including an increase in the electron density observed at the cytoplasmic periphery (Fig. 5k). Since some Iba1^+^ cells displayed an ameboid-like microglial phenotype (Fig. 5g–j), we further investigated whether the differentiated cells possessed a phagocytic capacity by exposing them to latex beads. We found that Iba1^+^ ameboid-like microglia took up the latex beads (Fig. 5l), indicating that reactive brain PCs can produce functional, phagocytic microglia.

### Brain PCs acquire stem cell and microglial traits following ischemia/hypoxia

**Fig. 6:**
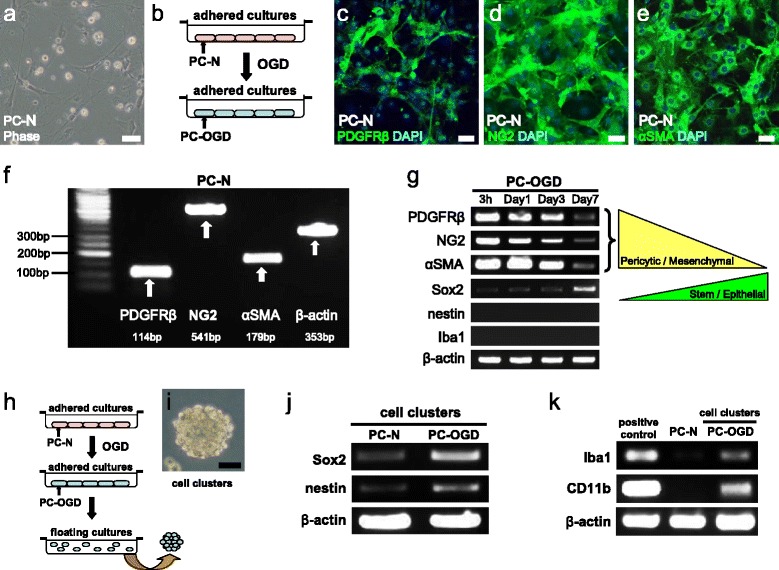
Oxygen/glucose deprivation induces brain PCs to acquire the traits of stem cells and microglia. Normal brain pericytes (PC-N) (**a**) were subjected to OGD (**b**). Immunohistochemistry showed that PC-N were positive for various pericytic markers, including PDGFRβ (PDGFRβ (**c** *green*), DAPI (**c** *blue*)), NG2 (NG2 (**d** *green*), DAPI (**d** *blue*)), and αSMA (αSMA (**e** *green*), DAPI (**e** *blue*)). PC-N expression of these pericytic markers was confirmed by RT-PCR (**f**). Following OGD treatment, PC-OGD showed a gradual decrease in pericytic/mesenchymal marker gene expression, including for NG2, PDGFRβ, and αSMA. In contrast, expression of the stem/epithelial cell marker Sox2 was upregulated (**g**). PC-OGD that formed cell clusters (**h**, **i**) showed strong expression of the stem cell markers Sox2 and nestin compared with that in the control cells (**j**). In addition, PC-OGD-associated cell clusters expressed Iba1 and CD11b (**k**). *Scale bars* = 50 μm (**a**, **c**, **d**, **e**, **i**) *PC-N* PCs cultured under normal/normoxia condition, *PC-OGD* PCs cultured under oxygen/glucose deprivation

Thus far, our data showed that brain PCs acquire specific stemness and microglial phenotypes in response to ischemia/hypoxia. However, the mechanism by which they acquired these traits remains unclear. To investigate this mechanism, commercially available, normal brain PCs (PC-N) (Fig. 6a) were incubated under OGD conditions (Fig. 6b) to mimic ischemia/hypoxia. Immunohistochemistry showed that PC-N express various pericytic markers, including PDGFRβ (Fig. 6c), NG2 (Fig. 6d), and αSMA (Fig. 6e). The RT-PCR results confirmed the PC-N expression of these markers (Fig. 6f). After OGD treatment (PC-OGD), pericytic/mesenchymal marker (PDGFRβ, NG2, and αSMA) expression was downregulated, while that of a stem/epithelial marker Sox2 was upregulated (Fig. 6g). These observations indicate that brain PCs develop stemness in a mesenchymal-epithelial transition (MET)-like manner.

However, PC-OGD did not express nestin and Iba1 (Fig. 6g). Thus, we further investigated whether PC-OGD acquire microglial phenotypes in parallel with an increase stem cell traits. In this study, PC-OGD were further incubated in floating cultures to develop stemness (Fig. 6h) as described previously [20]. Under these conditions, PC-OGD formed cell clusters (Fig. 6i) and they significantly increased their expression of stem cell markers, such as Sox2 and nestin (Fig. 6j). In contrast, control PC-N that formed cell clusters only weakly expressed these markers (Fig. 6j). Furthermore, we found that the PC-OGD that formed cell clusters expressed significantly elevated amounts of the microglial markers Iba1 and CD11b (Fig. 6k). These results suggest that brain PCs develop stemness following ischemia/hypoxia, thereby acquiring microglial phenotypes.

## Discussion

The multipotent stem cell activity of PCs, which allows them to differentiate into various cell types, including adipocytes, osteoblasts, chondrocytes, neural cells, and vascular cells, has been well documented [16, 25, 26, 37–43]. Previous reports have shown that PCs can differentiate into immune cells such as dendritic cells [44] and macrophage-like cells [45]. However, the ability of PCs to produce microglia has remained unclear because most reported studies based their investigation on ultrastructural findings alone [17–19, 23]. Although a recent report showed that brain PCs acquire a microglial phenotype after ischemia [36], the precise mechanism was not elucidated. The current study clearly demonstrated that brain PCs acquire multipotent VSC activity following ischemic stroke and can therefore produce functional microglia.

VSCs are capable of differentiating into multiple cell lineages [9, 14, 46]. Although the precise traits of these cells remain unclear, PCs located around capillaries are strong candidate VSCs, as are APCs located around larger vessels [9]. APCs are Sca1^+^, multipotent stem/progenitor cells that localize in the adventitia of blood vessels [47]. Similar to the traits of multipotent PCs [20], APCs exhibit the potential for multi-lineage differentiation into various cell populations, including adipocytes, osteoblasts, chondrocytes, myocytes, neural cells, and vascular cells [10–12, 48]. In addition, a recent study by Psaltis and colleagues showed that APCs, but not bone marrow cells, give rise to macrophages that co-express αSMA [13, 49]. Combined with the present results showing that αSMA was predominantly present at perivascular cells around large vessels, these findings suggest that APCs rather than PCs have the potential to produce macrophages. Furthermore, our current study showed that Iba1^+^ microglia expressed PDGFRβ but not αSMA, suggesting that PCs rather than APCs have the potential to produce microglia. The precise relationship between APCs and PCs remains unclear. However, since APCs and PCs both express several markers and display similar traits [14], both are likely to be multipotent VSCs that can produce cells of the microglia/macrophage lineage.

Consistent with our previous studies [20, 50], the current study demonstrated that PCs derived from the post-ischemic brain expressed various stem/undifferentiated cell markers as well as multipotency. Why do reactive PCs acquire multipotency following an ischemic stroke? Under normal conditions, PCs are quiescent cells that cycle slowly. However, upon stimulation, PCs proliferate, migrate, and differentiate into various cell types [25, 26, 51]. These characteristics suggest that PCs alter their phenotypes under pathologic conditions. In support of this concept, our previous and current studies demonstrated that brain PCs cultured under conditions of OGD and in a MET-like manner can be reprogrammed to become multipotent stem cells that express various stem/undifferentiated cell markers, including nestin, c-myc, Klf4, and Sox2 [20, 50]. These findings indicate that the properties of PCs under normal and pathological conditions are completely distinct and that reactive, but not quiescent, PCs are likely multipotent VSCs that can produce cells of various lineages.

In the present study, we found that PC-OGD that formed cell clusters further increased their expression of stem cell markers, such as Sox2 and nestin. In addition, PDGFRβ^+^ iPCs showed that pericytic markers were predominantly expressed in the peripheral zones of cell clusters but not in the cores. Although we do not know the exact reason for this phenomenon, the current study showed that pericytic marker expression was downregulated during MET that occurred following ischemia/hypoxia [20]. Therefore, MET most likely occurs within the hypoxic cores of cell clusters rather than in the peripheral zones.

Previous studies have shown that within brains under pathologic conditions, the pericytic marker NG2 was expressed in microglia determined to have multipotency [22, 52]. Consistent with these reports, we found that some NG2^+^ cells within ischemic areas expressed the microglial marker Iba1 (data not shown). Although it remains unclear whether NG2^+^ PCs can transform into microglia under these conditions, the present study showed that reactive PCs expressing PDGFRβ acquire stemness and can produce microglia. These results indicate that it is possible that some NG2^+^ microglia originate from PCs following injury. Together, these finding suggest that reactive PCs acquire not only stemness but also hematopoietic potential since microglia/macrophage-like cells have been reported to be derived from hematopoietic lineage cells, including hematopoietic stem cells [53, 54]. Why do reactive PCs acquire hematopoietic potential? Although adult brain PCs lack angiogenic properties under normal conditions, multipotent PCs do exhibit vasculogenic traits [40, 43, 55]. In addition, APCs expressed both PC (PDGFRβ, NG2) and hematopoietic stem cell (CD34) markers [40, 43, 55]. Furthermore, we recently demonstrated that following ischemia/hypoxia, adult brain PCs display a complex angioblastic phenotype that includes the expression of various hematopoietic stem cell markers such as CD34 and CD144 in addition to their original mesenchymal properties [20]. These cells acquired angioblastic traits along with enhanced expression of pluripotent markers such as Klf4 [20], which promotes angioblastic lineage reprogramming [56]. Combined with the finding that PDGFRβ is expressed in early hematopoietic precursors during development [57], these findings suggest that ischemia/hypoxia may convert normal PCs into reactive PCs with a mesenchymoangioblastic phenotype, which is typically observed in immature PCs during development [57, 58]. Nevertheless, the precise traits and subtypes of multipotent PCs with hematopoietic potential should be determined, ideally through future studies that include pericyte genetic lineage labeling experiments.

## Conclusions

We have demonstrated that reactive PCs give rise to microglia following ischemic stroke, suggesting that PCs play an important role in mediating inflammation under pathological conditions. Since reactive PCs can acquire multipotent stem cell activity and thus can differentiate into neural and vascular cells [20], our results indicate that brain PCs, a key component of the neurovascular unit that comprises neural cells, vascular cells, and microglia [59], can produce all components of this functional unit in response to ischemia/hypoxia. As the neurovascular unit serves as the minimal functional unit in the CNS, reactive PCs could potentially be targeted to regulate regeneration following CNS injury.

## Additional files

Additional file 1: Supplemental information. Methods for Additional files (2-4) are available in Supplemental methods of this file. (DOCX 56.8kb) Additional file 2: Figure S1. Localization and characterization of Iba1^+^ microglia after ischemic stroke. Immunohistochemical localization of Iba1 using the DAB reaction (A–L). In sham-operated mice, resting Iba1^+^ microglia showing a ramified morphology were observed in the MCA areas of the cortex (A–C). On post-ischemia day 3, many Iba1^+^ microglia were observed in peri-ischemic areas, with some cells exhibiting an ameboid-like, activated microglial morphology (D–F). On days 5 (G–I) and 7 after ischemia (J–L), most of the Iba1^+^ microglia exhibited an ameboid morphology and were localized in and around the ischemic areas. The numbers of Iba1^+^ cells in the ischemic core and peri-ischemic areas are indicated (M). The populations of ramified- or ameboid-like Iba1^+^ microglia in the ischemic core and peri-ischemic areas are shown (N). Scale bars = 100 μm (B, E, H, K) and 50 μm (C, F, I, L). Abbreviations: DAB, diaminobenzidine; Iba1, ionized calcium binding adaptor molecule 1; MCA, middle cerebral artery. (PDF 543kb) Additional file 3: Figure S2. PDGFRβ^+^ iPCs express the microglial marker CD206. On post-stroke day 3, some PDGFRβ^+^ cells within ischemic areas represent the microglial marker CD206 (A–D) (PDGFRβ (B, C: red), CD206 (B, D: green), DAPI (B–D: blue)) (arrows). Scale bars = 20 μm (B). Abbreviations: PDGFRβ, platelet-derived growth factor receptor-β. (PDF 209kb) Additional file 4: Figure S3. Localization and characterization of CD68^+^ cells following ischemia. CD68^+^ cells were rarely observed at the ischemic core and peri-ischemic areas on post-stroke day 3 (A–D) (Iba1 (B, C: red), CD68 (B, D: green), DAPI (B–D: blue)). Although only a small number of CD68^+^ cells were observed in these areas, on post-stroke day 5 (E–H) (Iba1 (F, G: red), CD68 (F, H: green), DAPI (F–H: blue)) and 7 (I–L) (Iba1 (J, K: red), CD68 (J, L: green), DAPI (J–L: blue)), some of them expressed Iba1 (arrows). However, these CD68^+^ cells rarely expressed PDGFRβ at post-stroke day 5 (M–P) (PDGFRβ (N, O: red), CD68 (N, P: green), DAPI (N–P: blue)) or day 7 (Q–T) (PDGFRβ (R, S: red), CD68 (R, T: green), DAPI (R–T: blue)). Scale bars = 20 μm (B, F, J, N, R). Abbreviations: Iba1, ionized calcium binding adaptor molecule 1; PDGFRβ, platelet-derived growth factor receptor-β. (PDF 398kb)

## Abbreviations

APCs: adventitial progenitor cells
CNS: central nervous system
Iba1: ionized calcium binding adaptor molecule 1
MACS: magnetic cell sorting
MCA: middle cerebral artery
MET: mesenchymal-epithelial transition
MHC: major histocompatibility complex
NCM: neural-conditioned medium
PCs: pericytes
PDGFRβ: platelet-derived growth factor receptor-β
VSCs: vascular stem cells
