# Supplementary material for: Brain pericytes serve as microglia-generating multipotent vascular stem cells following ischemic stroke
Source: J Neuroinflammation. 2016 Mar 7;13:57. doi: 10.1186/s12974-016-0523-9 (PMC4782566; doi:10.1186/s12974-016-0523-9)
Supplement: Additional file 1: Supplemental information. — Methods for Additional files (2-4) are available in Supplemental methods of this file. (DOCX 56.8kb) [file 12974_2016_523_MOESM1_ESM.docx]

**Supplemental Information**

**Brain pericytes serve as microglia-generating multipotent vascular stem cells following ischemic stroke**

Rika Sakuma^1^, Maiko Kawahara^1,2^, Akiko Nakano-Doi^1^,

Ai Takahashi^1,2^, Yasue Tanaka^1,3^, Aya Narita^1^, Sachi Kuwahara-Otani^5^, Tetsu Hayakawa^4^, Hideshi Yagi^5^, Tomohiro Matsuyama^1^, Takayuki Nakagomi^1#^

^1^Institute for Advanced Medical Sciences, ^3^Department of Neurosurgery, ^4^Laboratory of Tumor Immunology and Cell Therapy, ^5^Department of Anatomy and Neuroscience, Hyogo College of Medicine, Nishinomiya, Hyogo, Japan

^2^Graduate School of Science and Technology, Kwansei Gakuin University, Sanda, Hyogo, Japan

**^#^Correspondence:** Takayuki Nakagomi, M.D., Ph.D.,

Institute for Advanced Medical Sciences*, Hyogo College of Medicine,*

1-1 Mukogawacho, Nishinomiya, Hyogo, 663-8501, Japan.

Telephone: +81-798-45-6821;

Fax: +81-798-45-6823;

E-mail: nakagomi@hyo-med.ac.jp

**Supplemental Methods**

**Immunohistochemistry**

Coronal brain sections were prepared and subjected to immunohistochemistry as described previously [1-4]. In brief, mice were anesthetized with sodium pentobarbital and perfused transcardially with 4% paraformaldehyde on days 3, 5, and 7 after stroke. Perfused brains were removed, cryoprotected in 30% sucrose, and cut on a cryostat. Tissue sections were labeled with antibodies against ionized calcium binding adaptor molecule 1 (Iba1; Abcam, Cambridge, UK), PDGFRβ (Santa Cruz Biotechnology, Santa Cruz, CA, USA), CD206 (R&D Systems), or CD68 (Abcam). Primary antibodies were visualized using Alexa Fluor 488- or 555-conjugated secondary antibodies (Molecular Probes, Eugene, OR, USA). Nuclei were counterstained with 4’,6-diamidino-2-phenylindole (DAPI; Kirkegaard & Perry Laboratories, Inc., **Gaithersburg, MD, USA)**. Antibody-labeled brain sections were imaged using a confocal laser microscope (LSM780; Carl Zeiss, Jena, Germany). Additional brain sections were immunostained using peroxidase-conjugated secondary antibodies and the diaminobenzidine (DAB) reaction for visualization. The numbers of Iba1^+^ cells localized to the ischemic core and peri-ischemic areas (a total of 30 data points, 10 points/section [n = 3]) were analyzed and subjected to a semi-quantitative analysis as described [3, 5].

**References**

1. Nakagomi T, Taguchi A, Fujimori Y, Saino O, Nakano-Doi A, Kubo S, Gotoh A, Soma T, Yoshikawa H, Nishizaki T, et al: **Isolation and characterization of neural stem/progenitor cells from post-stroke cerebral cortex in mice.** *Eur J Neurosci* 2009, **29:**1842-1852.

2. Nakagomi N, Nakagomi T, Kubo S, Nakano-Doi A, Saino O, Takata M, Yoshikawa H, Stern DM, Matsuyama T, Taguchi A: **Endothelial cells support survival, proliferation, and neuronal differentiation of transplanted adult ischemia-induced neural stem/progenitor cells after cerebral infarction.** *Stem Cells* 2009, **27:**2185-2195.

3. Nakano-Doi A, Nakagomi T, Fujikawa M, Nakagomi N, Kubo S, Lu S, Yoshikawa H, Soma T, Taguchi A, Matsuyama T: **Bone marrow mononuclear cells promote proliferation of endogenous neural stem cells through vascular niches after cerebral infarction.** *Stem Cells* 2010, **28:**1292-1302.

4. Nakagomi T, Molnar Z, Nakano-Doi A, Taguchi A, Saino O, Kubo S, Clausen M, Yoshikawa H, Nakagomi N, Matsuyama T: **Ischemia-induced neural stem/progenitor cells in the pia mater following cortical infarction.** *Stem Cells Dev* 2011, **20:**2037-2051.

5. Saino O, Taguchi A, Nakagomi T, Nakano-Doi A, Kashiwamura S, Doe N, Nakagomi N, Soma T, Yoshikawa H, Stern DM, et al: **Immunodeficiency reduces neural stem/progenitor cell apoptosis and enhances neurogenesis in the cerebral cortex after stroke.** *J Neurosci Res* 2010, **88:**2385-2397.
