# Supplementary figures and images for: Brain pericytes serve as microglia-generating multipotent vascular stem cells following ischemic stroke
Source: J Neuroinflammation. 2016 Mar 7;13:57. doi: 10.1186/s12974-016-0523-9 (PMC4782566; doi:10.1186/s12974-016-0523-9)

# Supplementary Figure 1

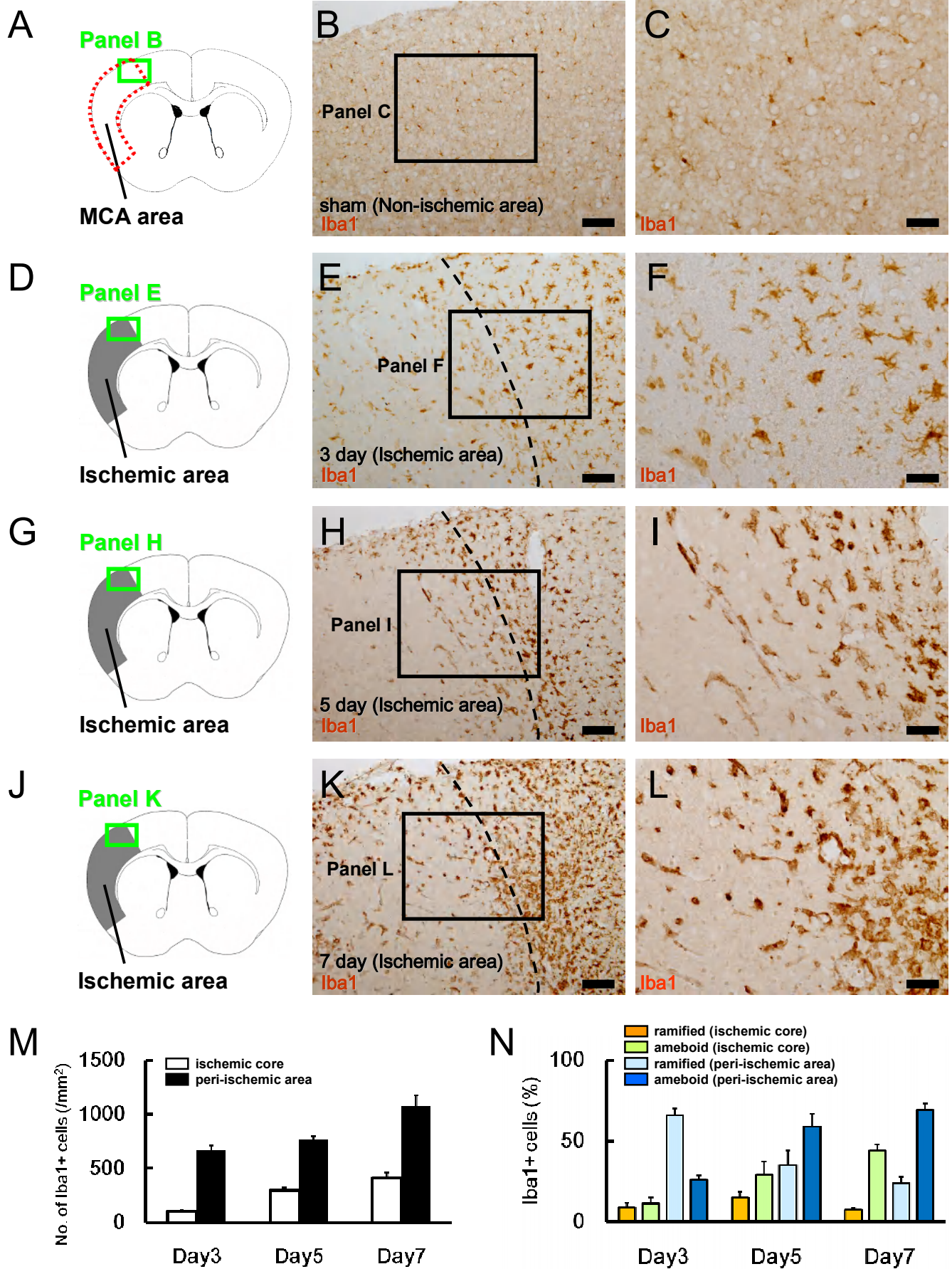

Supplement: Additional file 2: Figure S1. — Localization and characterization of Iba1+ microglia after ischemic stroke. Immunohistochemical localization of Iba1 using the DAB reaction (A–L). In sham-operated mice, resting Iba1+ microglia showing a ramified morphology were observed in the MCA areas of the cortex (A–C). On post-ischemia day 3, many Iba1+ microglia were observed in peri-ischemic areas, with some cells exhibiting an ameboid-like, activated microglial morphology (D–F). On days 5 (G–I) and 7 after ischemia (J–L), most of the Iba1+ microglia exhibited an ameboid morphology and were localized in and around the ischemic areas. The numbers of Iba1+ cells in the ischemic core and peri-ischemic areas are indicated (M). The populations of ramified- or ameboid-like Iba1+ microglia in the ischemic core and peri-ischemic areas are shown (N). Scale bars = 100 μm (B, E, H, K) and 50 μm (C, F, I, L). Abbreviations: DAB, diaminobenzidine; Iba1, ionized calcium binding adaptor molecule 1; MCA, middle cerebral artery. (PDF 543kb) [file 12974_2016_523_MOESM2_ESM.pdf]

## Supplementary Figure 2

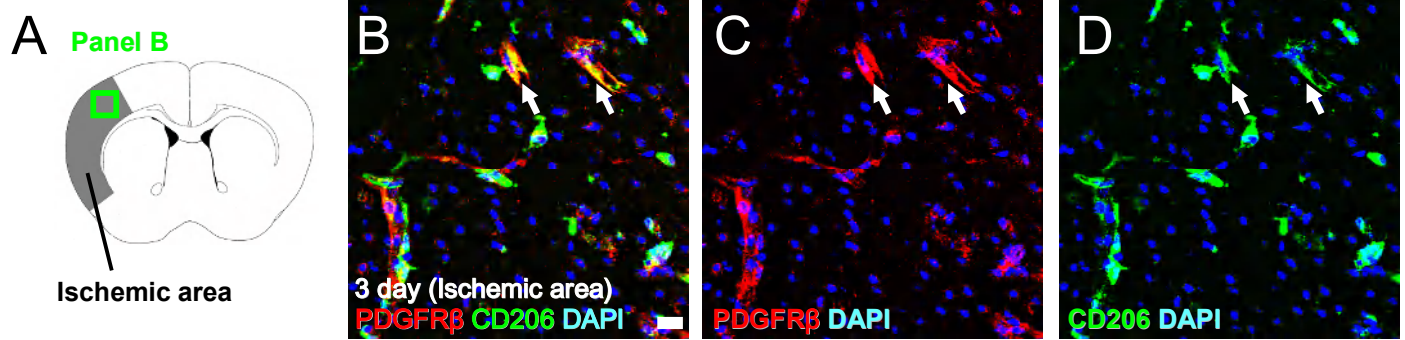

Supplement: Additional file 3: Figure S2. — PDGFRβ+ iPCs express the microglial marker CD206. On post-stroke day 3, some PDGFRβ+ cells within ischemic areas represent the microglial marker CD206 (A–D) (PDGFRβ (B, C: red), CD206 (B, D: green), DAPI (B–D: blue)) (arrows). Scale bars = 20 μm (B). Abbreviations: PDGFRβ, platelet-derived growth factor receptor-β. (PDF 209kb) [file 12974_2016_523_MOESM3_ESM.pdf]

## Supplementary Figure 3

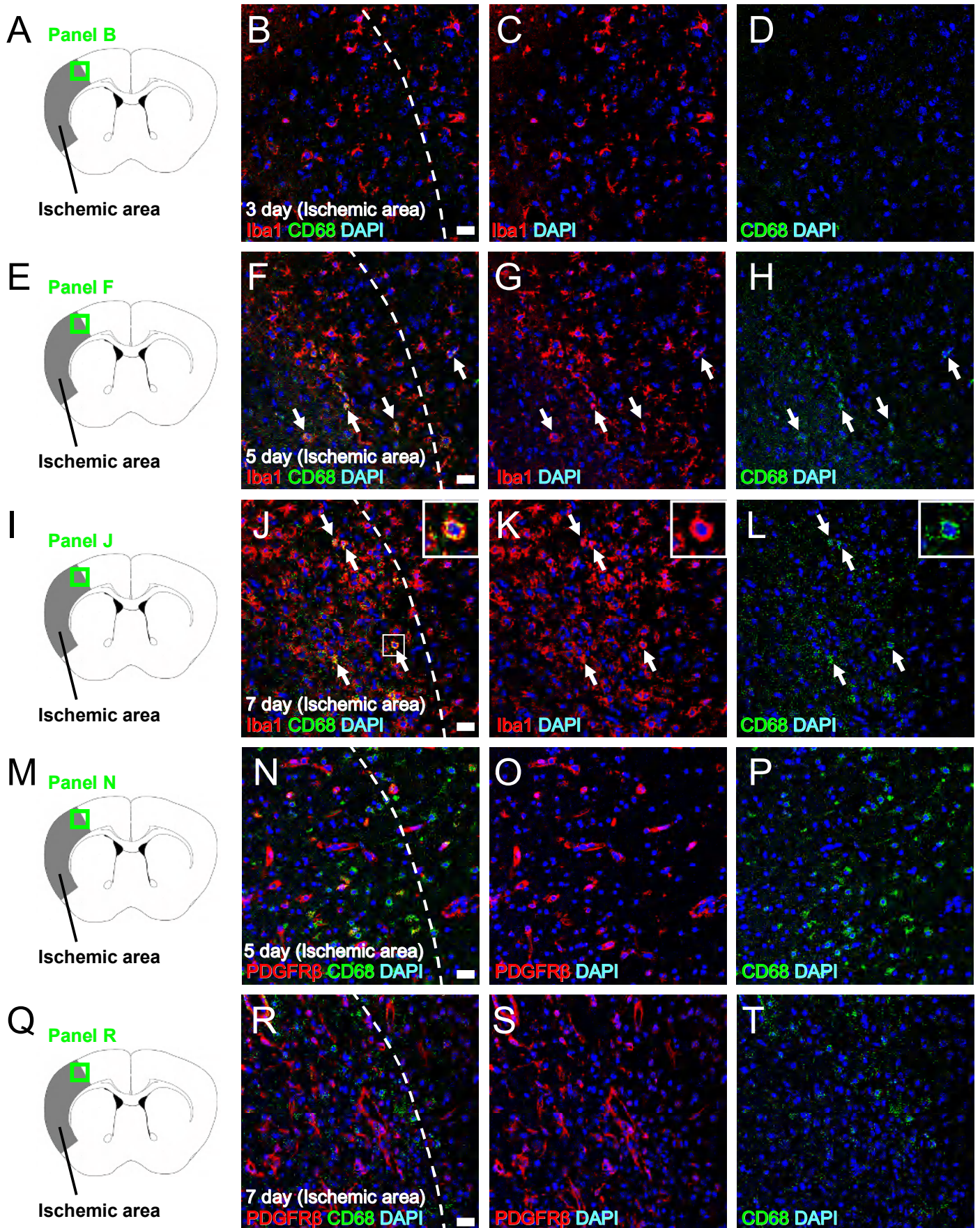

Supplement: Additional file 4: Figure S3. — Localization and characterization of CD68+ cells following ischemia. CD68+ cells were rarely observed at the ischemic core and peri-ischemic areas on post-stroke day 3 (A–D) (Iba1 (B, C: red), CD68 (B, D: green), DAPI (B–D: blue)). Although only a small number of CD68+ cells were observed in these areas, on post-stroke day 5 (E–H) (Iba1 (F, G: red), CD68 (F, H: green), DAPI (F–H: blue)) and 7 (I–L) (Iba1 (J, K: red), CD68 (J, L: green), DAPI (J–L: blue)), some of them expressed Iba1 (arrows). However, these CD68+ cells rarely expressed PDGFRβ at post-stroke day 5 (M–P) (PDGFRβ (N, O: red), CD68 (N, P: green), DAPI (N–P: blue)) or day 7 (Q–T) (PDGFRβ (R, S: red), CD68 (R, T: green), DAPI (R–T: blue)). Scale bars = 20 μm (B, F, J, N, R). Abbreviations: Iba1, ionized calcium binding adaptor molecule 1; PDGFRβ, platelet-derived growth factor receptor-β. (PDF 398kb) [file 12974_2016_523_MOESM4_ESM.pdf]
